# Tubular immaturity causes erythropoietin-deficiency anemia of prematurity in preterm neonates

**DOI:** 10.1038/s41598-018-22791-y

**Published:** 2018-03-13

**Authors:** Nariaki Asada

**Affiliations:** 1 Department of Pediatrics, Keio University School of Medicine, Tokyo, Japan; 2 Department of Pediatrics, SUBARU health insurance society Ota Memorial Hospital, Gunma, Japan

## Abstract

Kidneys are physiologically hypoxic due to huge oxygen consumption for tubular reabsorption. The physiological hypoxia makes the kidney an appropriate organ for sensitively detecting oxygen levels and producing erythropoietin (EPO). In preterm neonates, immature kidneys cannot produce sufficient EPO, which results in anemia of prematurity (AOP). The cause of EPO insufficiency in AOP has been unclear, therefore current therapeutic options are transfusion and injection of recombinant human EPO. This report shows that the cause of insufficient EPO production in AOP is elevated renal oxygen levels due to poor oxygen consumption by immature tubules. Neonatal mice with AOP showed low tubular transporter expression and elevated renal oxygen levels compared with those without AOP. Enhancing transporter expression in AOP mice induced renal hypoxia and EPO production. In preterm neonates, red blood cell counts, hemoglobin levels, and hematocrit levels correlated with tubular function, but not with serum creatinine, gestational age, or birth weight. Furthermore, pharmacological upregulation of hypoxia signaling ameliorated AOP in mice. These data suggest that tubular maturation with increased oxygen consumption is required for renal EPO production.

## Introduction

Erythropoietin (EPO), an essential hormone for red blood cell production, is mainly produced in the liver before birth and in the kidney after birth^1^. EPO is regulated in an oxygen-dependent manner by hypoxia inducible factor (HIF)^1^. Under hypoxic conditions, HIF proteins become stable and upregulate downstream genes including *EPO*. The kidney is a hypoxic organ because of huge oxygen consumption for tubular reabsorption^2,3^. The physiological hypoxia makes the kidney an appropriate organ for sensitively detecting oxygen levels and producing EPO.

EPO-deficiency anemia occurs in chronic kidney disease (CKD) patients and in preterm neonates. In CKD, renal anemia occurs since EPO-producing fibroblasts transdifferentiate into myofibroblasts in response to injuries^1,4^. On the other hand, preterm neonates develop another type of EPO-deficiency anemia, which is known as anemia of prematurity (AOP)^5^. Although AOP is also contributed by repetitive phlebotomies or iron deficiency, the primary cause is impaired ability to increase EPO production in immature kidneys. AOP occurs when neonates are born before 34 weeks of gestation^5^. Since nephrogenesis is not completed until 34 weeks of gestation, renal function, especially tubular function, of preterm neonates with AOP is usually so immature that body fluid, electrolytes, amino acids, glucose, and low molecular proteins are lost into urine^6–8^. Although it has been speculated that EPO insufficiency in AOP is also attributable to some kind of immaturity of the kidney, the precise mechanism remains unclear. Current therapeutic options are, therefore, transfusion and injection of recombinant human EPO.

This report shows that tubular maturation is required for the initiation of postnatal EPO production in the kidney. Neonatal mice with AOP showed low expression of tubular transporters, poor oxygen consumption rate, and elevated renal oxygen levels. Enhancing tubular transporter expression was sufficient to induce renal hypoxia and EPO production in AOP mice. In a clinical study of preterm neonates, red blood cell counts, hemoglobin levels, and hematocrit levels correlated with tubular function, but not with serum creatinine, gestational age, or birth weight, suggesting that tubular immaturity is important for the development of AOP in human. Furthermore, enhancing hypoxia signaling with prolyl hydroxylase inhibitor (PHDi) was shown to ameliorate AOP. These findings provide novel insights into the mechanisms of postnatal EPO production in the kidney and clues to develop therapeutic approaches for AOP.

## Results

### Neonatal mice show impaired EPO production similar to AOP

**Figure 1:**
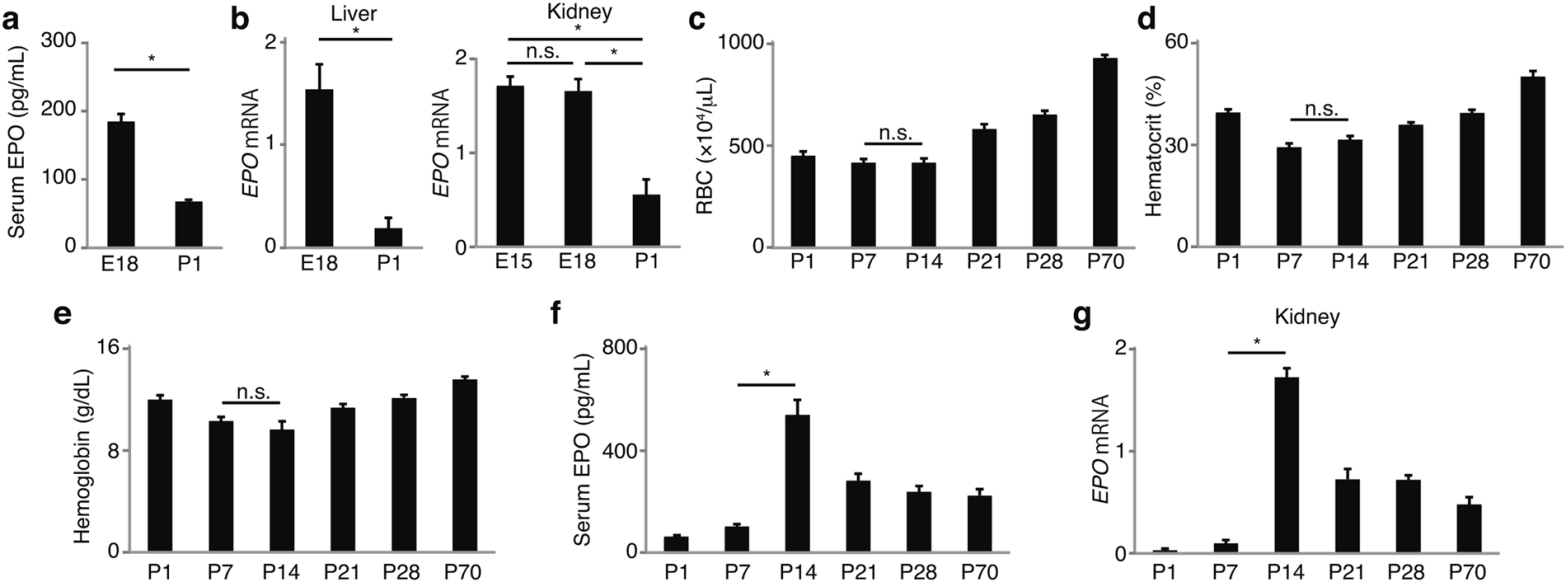
Neonatal mice show erythropoietin (EPO)-deficiency anemia of prematurity. (**a**) Serum EPO decreased from E18 to P1 reflecting postnatal oxygenation (*n* = 6). (**b**) *EPO* mRNA also decreased after birth not only in the liver but in the kidney (*n* = 6). (**c**–**f**) Postnatal RBC counts, hematocrit levels, hemoglobin levels, and EPO. The same degree of anemia was observed at P7 and P14 (*n* = 6) (**c**–**e**). Regardless of the same degree of anemia, serum EPO and renal *EPO* mRNA at P7 were much lower than those at P14. (*n* = 6) (**f**,**g**). Error bars indicate SEM. *P < 0.05 by t-test or Tukey-Kramer test. RBC, red blood cells; n.s., not significant. In the graphs of mRNA expression, arbitrary unit is used for vertical axis.

Mice are physiologically born premature, and nephrogenesis and tubular maturation continue after birth for up to 7 days and several weeks, respectively^7–10^. To investigate whether mice develop AOP due to the physiological premature birth, perinatal EPO, red blood cell (RBC) counts, hematocrit levels, and hemoglobin levels were evaluated. Serum EPO and *EPO* mRNA in the liver and kidney decreased after birth, reflecting postnatal oxygenation (Fig. 1a,b). Neonatal mice showed anemia compared with adult mice (Fig. 1c–e). Regardless of the same degree of anemia, both serum EPO levels and renal *EPO* mRNA expression at P7 were lower than those at P14 (Fig. 1f,g). The insufficient renal EPO production in anemia at P7 was considered to be the same as AOP. Hepatic *EPO* mRNA was elevated to the comparable levels at P7 and P14 reflecting the same degree of anemia (Supplementary Fig. 1).

### Immature kidneys are not hypoxic despite anemia

**Figure 2:**
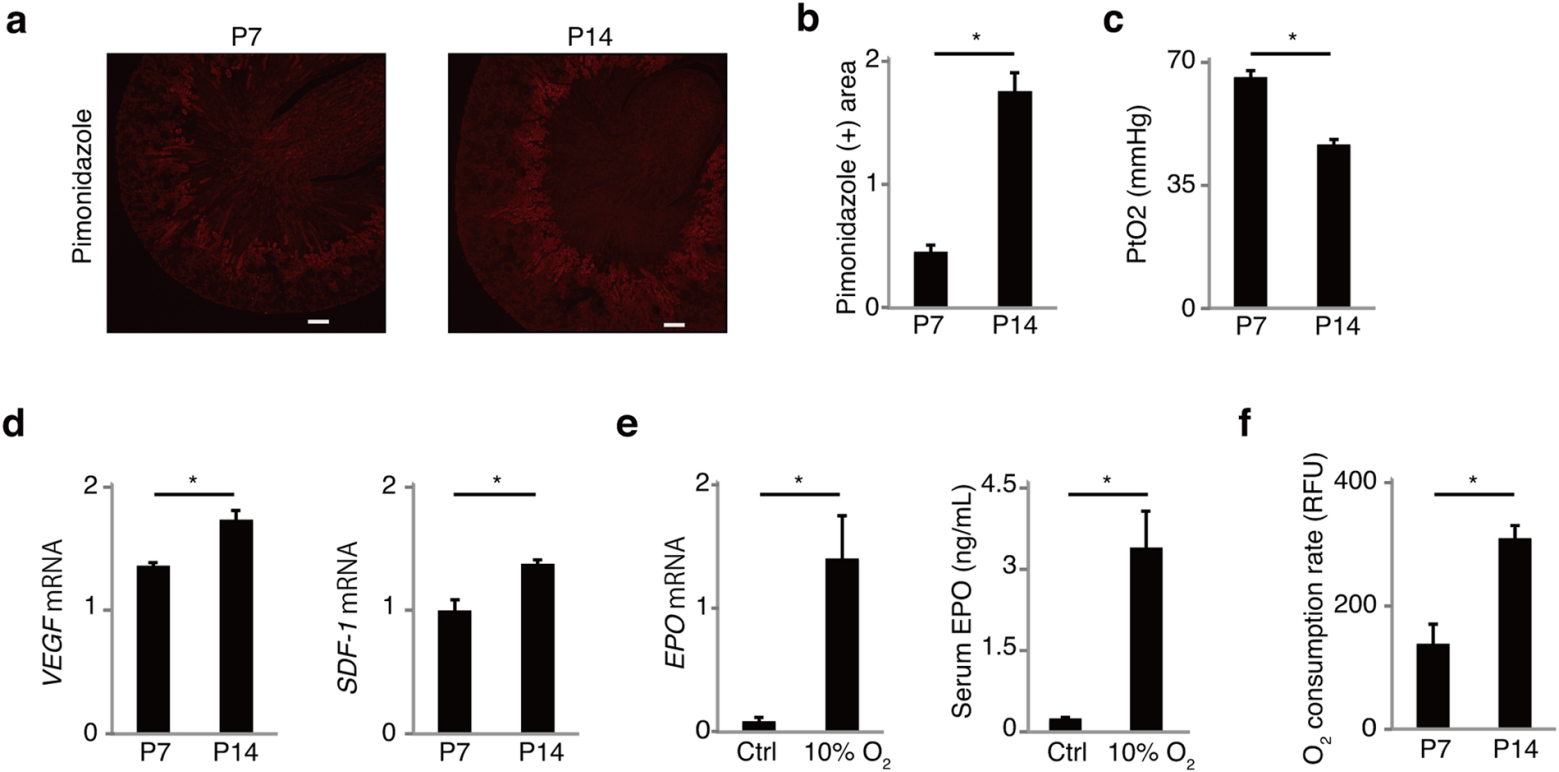
Renal hypoxia is insufficient in anemia of prematurity. (**a** and **b**) Pimonidazole-positive hypoxic area at P14 was broader than that at P7 (*n* = 6). (**c**) Renal tissue O_2_ partial pressure (PtO_2_) at P7 was higher than that at P14 (n = 6). (**d**) The mRNA expression of HIF-regulated genes, *VEGF* and *SDF-1*, increased from P7 to P14 (*n* = 4). (**e**) Neonatal kidneys at P7 were capable of increasing EPO production in response to hypoxia of 10% oxygen (*n* = 4). (**f**) Primary renal cells obtained from kidneys at P14 showed higher oxygen consumption rate than those at P7 (n = 6). Error bars indicate SEM. *P < 0.05 by t-test. n.s., not significant. Scale bars denote 100 micrometer. In the graphs of mRNA expression, arbitrary unit is used for vertical axis.

To know the cause of insufficient EPO production at P7, difference between the kidneys at P7 and P14 was investigated. In CKD, EPO production is impaired because of transdifferentiation of fibroblasts into alpha smooth muscle actin (αSMA)-positive myofibroblasts^1,4^. Since immature renal fibroblasts are also known to express myofibroblast markers^11^, αSMA was stained in the kidneys at P7 and P14. The expression of αSMA in renal fibroblasts was observed in medulla and inner cortex at both P7 and P14, denying the association of the myofibroblast phenotype with AOP (Supplementary Fig. 2a,b). Next, the degree of renal hypoxia was evaluated by immunostaining of pimonidazole, which accumulates in hypoxic area^12^. Both at P7 and P14, pimonidazole stained mainly at corticomedullary junction, where EPO is normally produced^4^. Kidneys at P7, however, showed limited pimonidazole-positive hypoxic area compared with P14, which suggests kidneys at P7 are less hypoxic than those at P14 (Fig. 2a,b). In addition, tissue pO_2_ was determined by Clark electrode, which measures oxygen concentration using catalytic platinum. The measurement of pO_2_ showed higher pO_2_ in the kidneys at P7 compared with P14 (Fig. 2c). Neither HIF1α nor HIF2α were detected by western blotting and immunostaining. There were no significant difference in the mRNA expression of *HIF1*α or *HIF2*α in the kidneys between P7 and P14 (Supplementary Fig. 2c). However, other HIF-regulated genes, *vascular endothelial growth factor* (*VEGF*) and *stromal cell-derived factor 1* (*SDF-1*) were elevated from P7 to P14 in the kidney, reflecting decreased renal oxygen levels (Fig. 2d).

The elevated *EPO* mRNA at E15 and E18 indicated that embryonic kidneys are already capable of increasing *EPO* expression in response to intrauterine hypoxia (Fig. 1b). To confirm that kidneys at P7 are also able to increase EPO production in the presence of hypoxia, mice are exposed to 10% oxygen. EPO sharply increased after 5 hours of the hypoxia (Fig. 2e). These data suggest that immature kidneys can increase EPO production in response to hypoxia signaling.

Next, to investigate the cause of the difference in renal oxygen levels, oxygen consumption rate (OCR) was compared between P7 and P14. OCR assay with primary kidney cells revealed that OCR at P14 is higher than that at P7 (Fig. 2f). Although capillary density is also known to be associated with renal oxygen levels^2^, immunostaining of endothelial marker CD31 showed that peritubular capillary density was comparable between P7 and P14 (Supplementary Fig. 2d,e). The expression of *CD31* mRNA in the kidney, which correlates with capillary density, was also comparable between P7 and P14 (Supplementary Fig. 2f).

### Tubular reabsorption is associated with renal hypoxia and EPO production in mice

**Figure 3:**
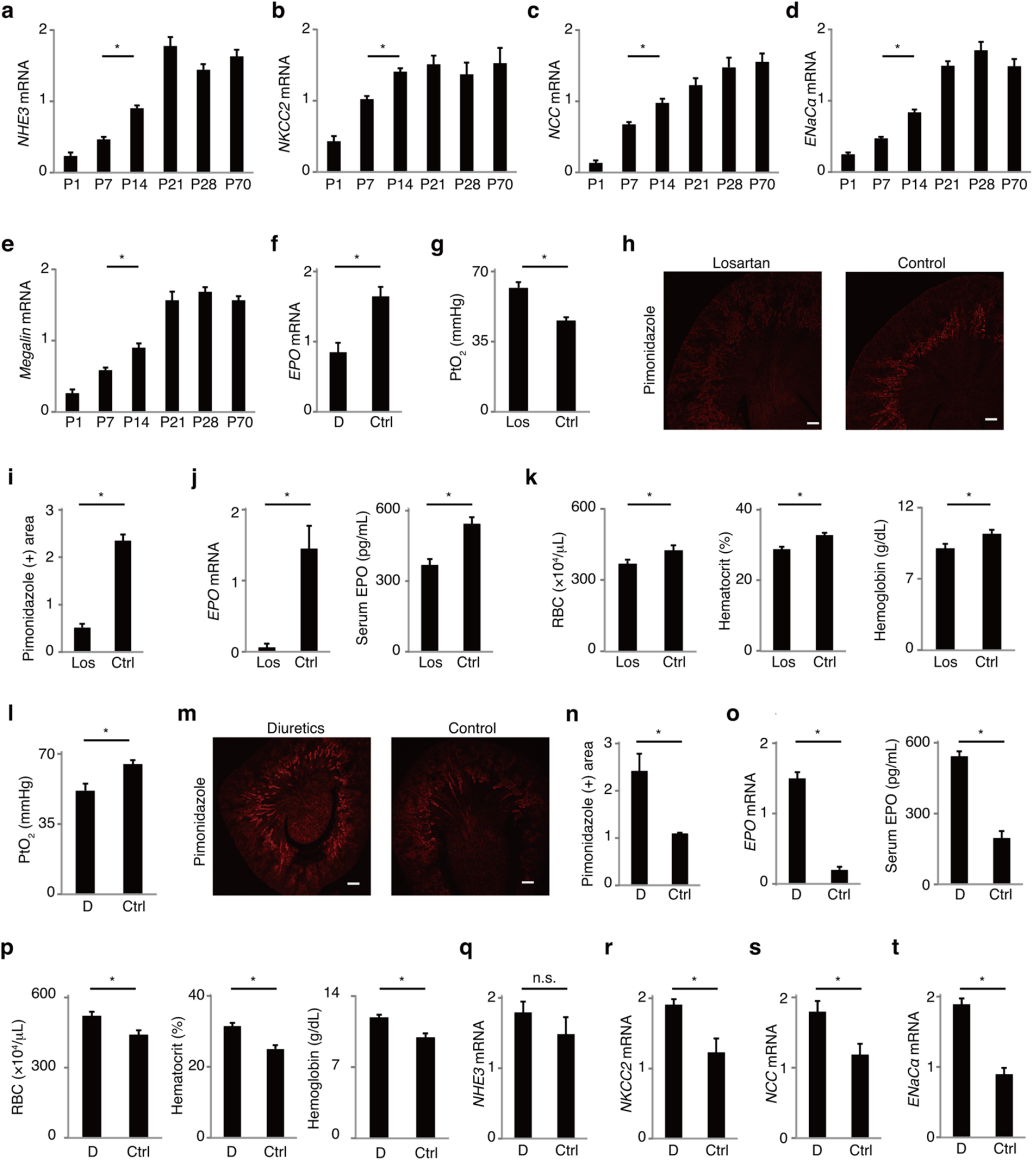
Association of tubular reabsorption with EPO in neonatal kidneys. (**a**–**e**) The expression of tubular transporters at P7 was lower than that at P14 (*n* = 6). (**f**–**k**) Inhibition of reabsorption results in reduced renal EPO production. (**f**) The expression of *EPO* mRNA decreased 90 minutes after the use of diuretics (combination of acetazolamide, furosemide, hydrochlorothiazide, and spironolactone) (*n* = 4). (**g**–**k**) Use of losartan, which reduces glomerular filtration and tubular reabsorption, elevated renal PtO_2_ (**g**), and reduced pimonidazole-positive hypoxic area (**h** and **i**), EPO levels (**j**), and RBC counts, hematocrit levels, and hemoglobin levels (**k**) (*n* = 4). (**l**–**t**) Enhancing reabsorption results in increased EPO production. Long-term use of diuretics (combination of acetazolamide and furosemide) with normal saline supplement from P3 lowered renal PtO_2_ (**l**), and increased pimonidazole-positive hypoxic area (**m** and **n**), EPO levels (**o**), RBC counts, hematocrit levels, and hemoglobin levels (**p**) at P7 compared with the control. The expression of sodium transporters was elevated in the diuretics group (**q**–**t**) (*n* = 4). Error bars indicate SEM. *P < 0.05 by t-test or Tukey-Kramer test. n.s., not significant. Scale bars denote 100 micrometer. In the graphs of mRNA expression, arbitrary unit is used for vertical axis. RBC, red blood cells; D, diuretics; Ctrl, control; Los, losartan.

Tubular oxygen consumption for reabsorption contributes to renal hypoxia in mature kidneys^2,3^. In preterm infants with AOP, however, tubules are too immature to maintain homeostasis^6^. These suggest that hypoxic environment is not developed in immature kidneys due to poor oxygen consumption by tubules. Therefore the association of tubular reabsorption with renal hypoxia and EPO production was evaluated in the neonatal kidneys. The mRNA expression of sodium transporters and endocytic receptor *Megalin* at P7 was lower than those at P14 and older ages (Fig. 3a–e). To confirm that tubular reabsorption is associated with the elevated EPO production at P14, reabsorption was suppressed pharmacologically. A combination of acetazolamide, furosemide, hydrochlorothiazide, and spironolactone reduced renal *EPO* expression 90 minutes after injection (Fig. 3f). To suppress tubular reabsorption chronically, angiotensin receptor blocker (ARB), which decreases tubular reabsorption through reducing glomerular filtrating rate and directly inhibiting sodium reabsorption at proximal tubules^2,13,14^, was used from P10 to P14. The use of losartan increased renal pO_2_ and reduced renal hypoxic area, EPO levels, red blood cell counts, hematocrit levels, and hemoglobin levels at P14 (Fig. 3g–k). The decrease of *EPO* expression by diuretics or losartan was also observed in adult kidneys (Supplementary Fig. 3a,b). Next, tubular reabsorption was enhanced to confirm that the cause of AOP at P7 is immature tubular function. It is reported that chronic use of diuretics leads to compensatory increased reabsorption, tubular hypertrophy, and rise in Na, K-ATPase activity in other tubule segments^15,16^. In neonatal rodents, chronic use of diuretics is also known to enhance transporter expression^10,17^. Chronic use of acetazolamide and furosemide from P3 lowered renal pO_2_ and increased renal hypoxic area, EPO levels, RBC counts, hematocrit levels, hemoglobin levels, and the expression of sodium transporters at P7 (Fig. 3l–t). Body weight was comparable between the 2 groups, denying that elevated RBC counts, hematocrit levels, and hemoglobin levels were caused by hemoconcentration due to dehydration (Supplementary Fig. 3c). Although administration of only furosemide also significantly increased the expression of *EPO* and *ENaCα*, the increase of EPO was smaller compared with the combination of acetazolamide and furosemide (Supplementary Fig. 3d–l). These data suggest that tubular reabsorption is closely associated with renal EPO production in neonatal mice.

### PHDi ameliorates AOP in mice

**Figure 4:**
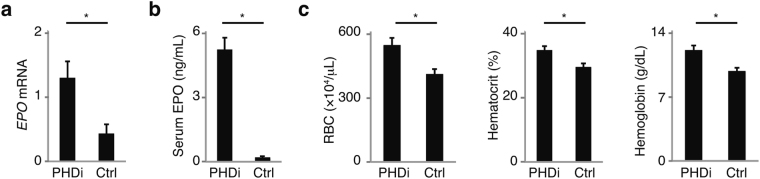
Prolyl hydroxylase inhibitor (PHDi) ameliorates anemia of prematurity. Administration of PHDi increased *EPO* mRNA (**a**), serum EPO levels (**b**), RBC counts, hematocrit levels, and hemoglobin levels (**c**) at P7 (*n* = 4). Error bars indicate SEM. *P < 0.05 by t-test. In the graph of mRNA expression, arbitrary unit is used for vertical axis. PHDi, prolyl hydroxylase inhibitor; Ctrl, control; RBC, red blood cells.

Considering the cause of AOP is insufficient hypoxia signaling, pharmacological upregulation of HIF signaling can be a therapeutic option. HIF protein is degraded by prolyl hydroxylase under normoxic conditions. Therefore inhibition of prolyl hydroxylase leads to stabilization of HIF protein and upregulation of HIF signaling^1^. Administration of PHDi actually increased renal *EPO* mRNA, serum EPO, red blood cell counts, hematocrit levels, and hemoglobin levels at P7 (Fig. 4a–c). These data suggest that enhancing hypoxia signaling is sufficient to ameliorate AOP.

### AOP is associated with tubular function in preterm neonates

**Figure 5:**
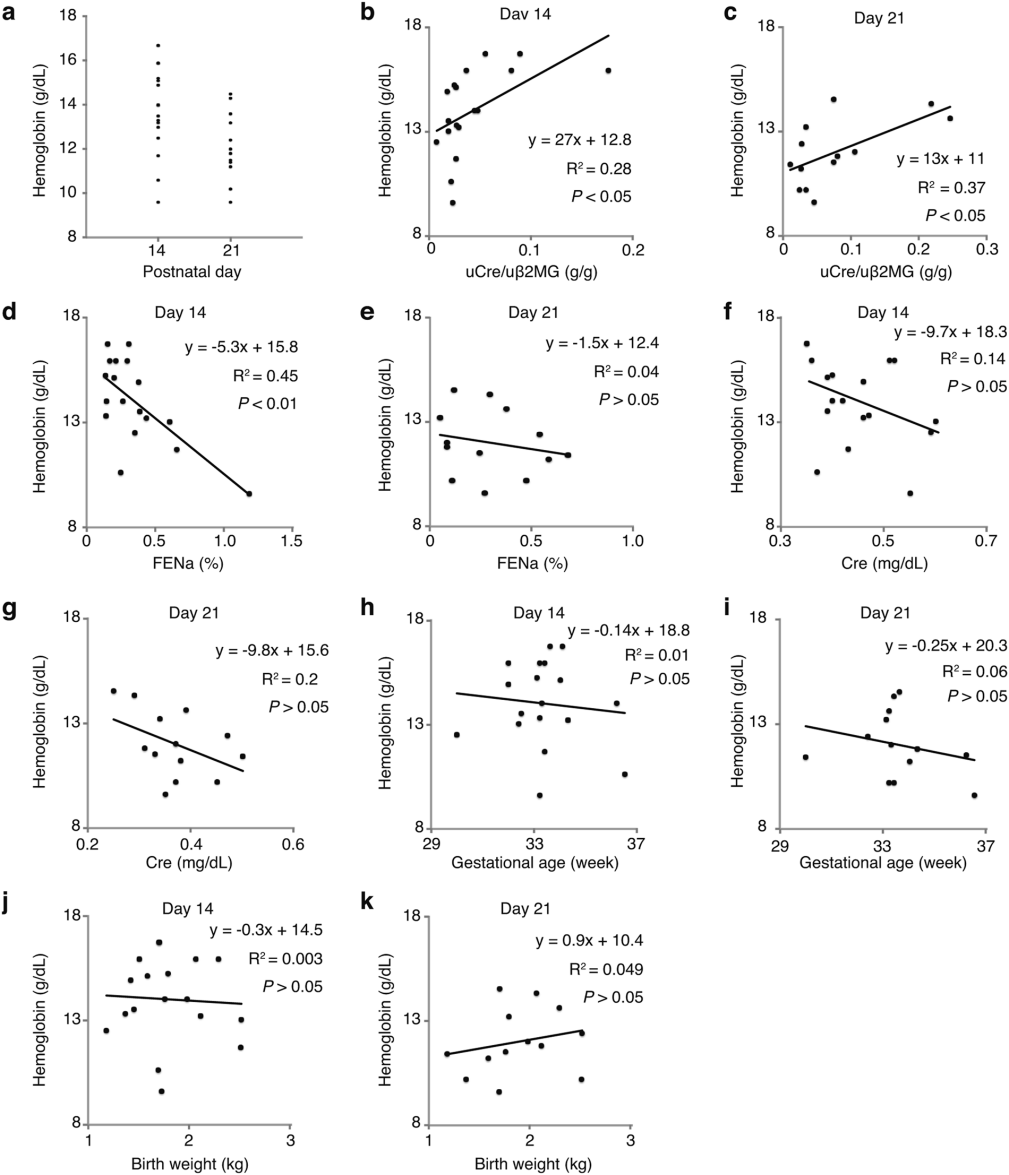
Tubular function correlates with hemoglobin levels in preterm neonates. (**a**) Hemoglobin levels of preterm neonates at age 14 days and 21 days. (**b** and **c**) Hemoglobin levels were significantly correlated with uCr/uβ2MG at days 14 and 21. (**d** and **e**) Hemoglobin levels were correlated with FENa at day 14, but not at day 21. (**f**–**k**) Hemoglobin levels were not correlated with serum creatinine (**f** and **g**), gestational age (**h** and **i**), or birth weight (**j** and **k**). Association between two variables was evaluated by Pearson correlation. *n* = 18 for day 14 and *n* = 13 for day 21. uCr/uβ2MG, urinary creatinine to beta 2-microglobulin ratio; FENa, fractional excretion of sodium; Cre, creatinine.

To investigate the association between tubular maturity and AOP in human, the relation of RBC counts, hemoglobin levels, and hematocrit levels with urinary creatinine to beta 2-microglobulin ratio (uCr/uβ2MG), fractional excretion of sodium (FENa), serum creatinine, gestational age, or birth weight were analyzed in preterm neonates at ages of 14 and 21 days, when AOP usually occurs^5^. uCr/uβ2MG and FENa were used as indicators of tubular maturity^18^. Characteristics of 18 patients enrolled are shown in Table 1. Hemoglobin levels ranged from 9.6 to 16.7 g/dL and from 9.6 to 14.5 g/dL at days 14 and 21, respectively (Fig. 5a). All of RBC counts, hemoglobin levels, and hematocrit levels were significantly correlated with uCr/uβ2MG at days 14 and 21 (Fig. 5b,c, Supplementary Figs 4a,b, 5a,b) and with FENa at day 14 but not at day 21 (Fig. 5d,e, Supplementary Figs 4c,d, 5c,d). However, neither RBC counts, hemoglobin levels, nor hematocrit levels were correlated with serum creatinine, gestational age, or birth weight (Fig. 5f–k, Supplementary Figs 4e–j, 5e–j). These data support that tubular immaturity is associated with AOP in preterm neonates.

**Table 1:** Patients characteristics.

	Median (range)
Gestational age (weeks)	33 (30–36.7)
Birth weight (g)	1711 (1180–2504)
Apgar score 1 min	7 (3–9)
Apgar score 5 min	9 (7–9)
RBC at day 14 (×10^4^/μL)	395 (273–459)
RBC at day 21 (×10^4^/μL)	345 (284–422)
Hb at day 14 (g/dL)	14 (9.6–16.7)
Hb at day 21 (g/dL)	11.8 (9.6–14.5)
Hematocrit at day 14 (%)	38.8 (27.5–47.5)
Hematocrit at day 21 (%)	33.0 (27.2–41.0)
Cre at day 14 (g/dL)	0.43 (0.35–0.6)
Cre at day 21 (g/dL)	0.37 (0.25–0.5)
uCr/uB2MG at day 14 (g/g)	0.0266 (0.0074–0.18)
uCr/uB2MG at day 21 (g/g)	0.0461 (0.011–0.25)
FENa at day 14 (%)	0.28 (0.14–1.2)
FENa at day 21 (%)	0.27 (0.049–0.67)

## Discussions

This is the first report to investigate the cause of EPO deficiency in AOP. This study showed that neonatal kidneys with AOP were not hypoxic regardless of anemia, therefore the lack of hypoxia signaling was considered to be a cause for the EPO deficiency. Although it has been known that tubular oxygen consumption contributes to renal hypoxia, the association between tubular function and EPO production remained unclear. This study showed that tubular function is closely associated with renal EPO production and that the maturation of tubules is necessary for the postnatal EPO production in the kidney.

Immaturity of renal EPO-producing cells has been speculated as a reason for the EPO deficiency in AOP. However, elevated *EPO* expression in response to intrauterine hypoxia was observed not only in the liver but also in the kidney, suggesting that EPO-producing cells in embryonic kidneys are already capable of enhancing EPO production in response to hypoxia (Fig. 1b). Actually, it is reported that EPO production is observed from very early stages of development. In mice, neural crest cells produce EPO for primitive erythropoiesis at E9^19^, and migrate into the kidneys by E13.5 to become renal EPO-producing fibroblasts^4^. In late embryonic stages, therefore, maturation of renal EPO-producing cells may not be as important as hypoxia signaling.

Tubular maturation was considered to be a major contributing factor for development of renal hypoxia. Although only expression of some transporters was examined in this article, other tubular maturation processes including increase in the surface area of brush border, in the number of mitochondria, and in the activity of Na^+^/K^+^-ATPase are also thought to contribute to renal hypoxia^7,20^. It is reported that excessive hypoxia signaling in tubules downregulates tubular oxygen consumption^21^ and lowers renal EPO production. Enhanced hypoxia signaling in mice kidneys at P14 might suppress tubular oxygen consumption and delay recovery from AOP.

Diuretics induced compensatory upregulation of transporter expression, renal hypoxia, and EPO production (Fig. 3l–t and Supplementary Fig. 3d–l). Therefore the use of diuretics may be beneficial for AOP, although there are risks of side effects. Interestingly, suppressing only NKCC2 transporter with furosemide was sufficient to ameliorate AOP (Supplementary Fig. 3d–l). In Bartter syndrome, in which *NKCC2* is mutated, patients with erythrocytosis were reported^22,23^. Elevated hematocrit levels in Bartter syndrome might be caused not only by hemoconcentration but by elevated EPO production.

The study of preterm neonates showed the correlation of red blood cell counts, hemoglobin levels, and hematocrit levels with tubular maturity but not with gestational age or glomerular function. uCr/uβ2MG was used as a tubular maturity marker since it showed better correlation than uβ2MG/uCr. The clinical data suggest that tubular immaturity is important for the development of AOP even in human. In the study, patients with acute kidney injury (AKI) or neonatal asphyxia of 5-minustes Apgar score <7 were not enrolled to avoid the effects of tubular injuries. Therefore very preterm neonates, who are at high risks of AKI and low Apgar score^8^, were not included, resulting in the small cohort. In very preterm neonates, AOP can be severe because of critically immature tubules and kidney injuries. However, analyzing the association between anemia and tubular function in very preterm neonates was difficult as they usually receive transfusion soon after birth.

This paper provided the first evidence for PHDi as a therapeutic option for AOP. PHDi has attracted interests as a potential treatment for renal anemia^1^. Although diseased kidneys usually become severely hypoxic, CKD patients develop renal anemia because of inflammatory signals, transdifferentiation of EPO-producing cells, and hypermethylation of *EPO*^2,4,24^. PHDi ameliorates renal anemia by excessively enhancing HIF signaling. On the other hand, the primary reason of AOP was thought to be insufficient hypoxia. Therefore, stabilizing HIF protein will be a more reasonable approach. There is a concern that PHDi may increase the risk of retinopathy of prematurity (ROP) by enhancing *VEGF* expression. Whether PHDi accelerates or ameliorates ROP may, however, depend on timing. In mice, PHDi is reported to prevent ROP if used in the early phase of the retinopathy^25^. Therefore, both ROP and AOP may be prevented if PHDi is started soon after birth. Before clinical application, further research regarding safety is needed since the influence of PHDi on organ development and maturation is still unclear. If PHDi is too toxic at this age, it cannot be an option at all and further research will be required to identify alternative strategies to target the same pathway.

This study has limitations. First, immunostaining and western blotting for HIF protein were technically impossible. Since it is reported that HIF can be detected only when kidneys are exposed to extremely hypoxic conditions including fatal anemia, use of carbon monoxide or cobaltous chloride, or renal artery clamping^26^, the expression of HIF protein in AOP was considered to be under detection limit even at P14. Second, the possibility that chronic use of ARB affected kidney development cannot be excluded. Since renin-angiotensin system is essential for kidney development, reduced expression of EPO might be partly due to impaired kidney development. Third, the cohort of clinical study was small. Further study with larger cohort is required.

In conclusion, the association between tubular function and renal EPO production has been demonstrated. The findings will help understand the mechanisms of EPO production in the kidney as well as EPO-deficiency anemia.

## Methods

### Mice

C56BL/6 mice were purchased from Sankyo Labo Service Corporation. All animal studies were approved by the Animal Experiment Committee of Keio University. All methods were performed in accordance with the relevant guidelines and regulations. In hypoxia experiments, mice were kept in cages with 10% oxygen and 90% nitrogen. Mice were sacrificed 5 hours later.

### Serum EPO measurement

Serum EPO was measured using Mouse Erythropoietin Quantikine ELISA kit (R&D MEP00B).

### RNA extraction, reverse transcription, real-time PCR

RNA was extracted from the kidney using TRIzol Reagent (Invitrogen). ReverTra Ace qPCR RT Master Mix (TOYOBO) was used for reverse transcription. RNA expression was evaluated by real-time PCR using StepOnePlus and Fast SYBR Green Master Mix (Applied Biosystems). Real-time PCR results were normalized with housekeeping gene 18 S rRNA.

### Primer sequence

EPO Fwd CATCTGCGACAGTCGAGTTCTG, Rev CACAACCCATCGTGACATTTTC: VEGF Fwd TACTGCTGTACCTCCACCTCCACCATG, Rev TCACTTCATGGGACTTCTGCTCT: SDF-1 Fwd ATGAACGCCAAGGTCGTGGTC, Rev GGTCTGTTGTGCTTACTTGTTT: NHE3 Fwd TGCCTTGGTGGTACTTCTGG, Rev TCGCTCCTCTTCACCTTCAG: NKCC2 Fwd GGCTTGATCTTTGCTTTTGC, Rev CCATCATTGAATCGCTCTCC: NCC Fwd CTTCGGCCACTGGCATTCTG, Rev GATGGCAAGGTAGGAGATGG: αENaC Fwd CATGCCTGGAGTCAACAATG, Rev CCATAAAAGCAGGCTCATCC: Megalin Fwd CAGTGGATTGGGTAGCAGGA, Rev GCTTGGGGTCAACAACGATA: HIF1α Fwd CCTGCACTGAATCAAGAGGTTGC, Rev CCATCAGAAGGACTTGCTGGCT, HIF2α Fwd GGACAGCAAGACTTTCCTGAGC, Rev GGTAGAACTCATAGGCAGAGCG: 18S rRNA Fwd CGCGGTTCTATTTTGTTGGT, Rev AGTCGGCATCGTTTATGGTC.

### Immuohistochemical analysis

Immunostaining was performed as described previously (5) using following antibodies: CD31 (Spring Bioscience) and αSMA (Sigma-Aldrich).

### Oxygen consumption rate assay

Kidneys at P7 and P14 were dissociated by incubation with dispase for 1 hour. Cells at a density of 4 × 10^6^/well were assayed for oxygen consumption rate using Extracellular O_2_ Consumption Assay kit (ab197243).

### Measurement of renal oxygen levels

PtO_2_ was measured using Clark electrode (BRC). After anesthesia and removal of renal capsule, electrode was inserted from the cortex to corticomedullary border. Mice were placed on a heat pad to avoid hypothermia during the measurement.

### Pimonidazole staining

Hypoxyprobe^TM^-1 Omni (COSMO BIO) was used to evaluate tissue hypoxic area. Mice were intraperitoneally injected with 60 mg/kg of pimonidazole and sacrificed 90 minutes later. Pimonidazole-positive area was evaluated by Image J.

### Pharmacological experiments

To suppress tubular reabsorption with diuretics, 20 mg/kg acetazolamide, 10 mg/kg furosemide, 10 mg/kg hydrochlorothiazide, and 10 mg/kg spironolactone were injected intraperitoneally. Kidneys were harvested 180 and 90 minutes after the injection in adult and neonatal mice, respectively. To suppress tubular reabsorption with losartan in neonatal mice, losartan (10 mg/kg/dose, 3 times/day) was injected from P10 to P14. To suppress tubular reabsorption with losartan in adult mice, 10 mg/kg losartan were administered intraperitoneally and kidneys were harvested 6 hours later. To upregulate tubular reabsorption, 10 mg/kg/dose acetazolamide and 5 mg/kg/dose furosemide in 20 mL/kg/dose normal saline or 5 mg/kg/dose furosemide in 20 mL/kg/dose normal saline were injected twice a day subcutaneously from P3 to P7. Normal saline were co-administered to avoid volume depletion and poor weight gain. Control group received only 20 mL/kg/dose normal saline twice a day from P3 to P7. For experiment with prolyl hydroxylase inhibitor, 50 mg/kg DMOG (ADQ A13998) was injected subcutaneously from P3 to P7. Mice were sacrificed 3 hours after the last injection.

### Clinical study

Preterm neonates less than 34 weeks of gestation or those less than 2000 gram of birth weight who were born in Ota Memorial Hospital between April in 2014 to March in 2015 were enrolled. Those who had neonatal asphyxia (Apgar score less than 7 at 5 minutes), AKI (increase in serum creatinine level of >0.3 mg/dl), and those who received transfusion were excluded. Blood test was performed only on days 0, 3, 7, 14, and 21 for patient management. Data for this study were collected on postnatal day 14 and 21. Data on days 14 and 21 were analyzed separately to avoid the influence of HbF. Data on day 21 was not collected when patients were discharged or received recombinant human EPO. The study was approved by the Institutional Review Board of Ota Memorial Hospital. Written informed consent for enrolling and evaluating hemoglobin, serum creatinine, uβ2MG, uCr, FENa was obtained from parents prior to inclusion in the study. Participants were identified by number. All methods were performed in accordance with the relevant guidelines and regulations.

### Statistics

Error bars indicate SEM. Difference between 2 groups were evaluated by t-test. Statistical comparisons among multiple groups were analyzed by Tukey-Kramer test. Association between two variables was evaluated by Pearson correlation. A P-value of less than 0.05 was considered statistically significant.

## Electronic supplementary material

Supplementary figures
