# Supplementary material for: Tubular immaturity causes erythropoietin-deficiency anemia of prematurity in preterm neonates
Source: Sci Rep. 2018 Mar 13;8:4448. doi: 10.1038/s41598-018-22791-y (PMC5849759; doi:10.1038/s41598-018-22791-y)
Supplement: Supplementary file 1 — Supplementary figures [file 41598_2018_22791_MOESM1_ESM.pdf]

Tubular immaturity causes erythropoietin-deficiency anemia of prematurity in preterm neonates

Nariaki Asada, MD [1, 2*]

Affiliations: 1. Department of Pediatrics, Keio University School of Medicine, Tokyo, Japan; 2. Department of Pediatrics, SUBARU health insurance society Ota Memorial Hospital, Gunma, Japan

***Corresponding author**: Nariaki Asada, Department of Pediatrics, Keio University School of Medicine, 35 Shinanomachi, Shinjuku-ku, Tokyo, 160-8582, Japan, phone: +81-3-3353-1211, FAX: +81-3-5379-1978; [secretgardenrei@gmail.com]

Supplementary figure 1

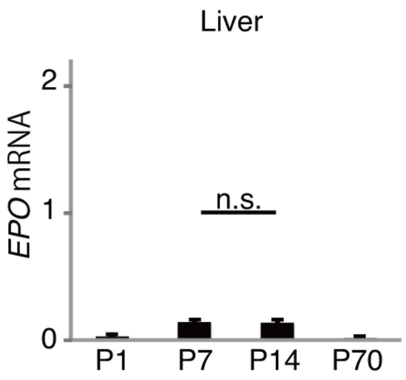

EPO mRNA in the liver increased to the same levels at P7 and P14 in response to the same degree of anemia (n = 4). Error bars indicate SEM. n.s., not significant by Tukey-Kramer test.

Supplementary figure 2

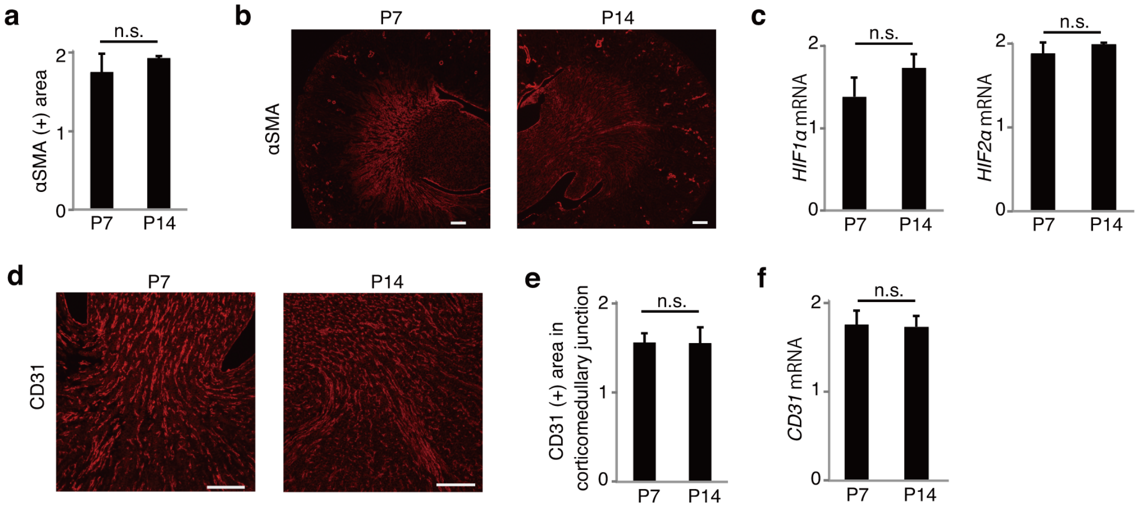

(**a** and **b**) The expression of myofibroblast marker, alpha-smooth muscle actin (αSMA), was observed not only in the vessels but in interstitium both at P7 and P14 (*n* = 4). (**c**) The mRNA expression of *HIF1α* and *HIF2α* in the kidney was comparable between P7 and 14 (*n* = 4). (**d**-**f**) Capillary density, evaluated by CD31-positive area (**d** and **e**) or by the expression of *CD31* mRNA (**f**) was comparable between P7 and P14 (*n* = 4). Error bars indicate SEM. n.s., not significant by t-test. Scale bars denote 100 micrometer.

Supplementary figure 3

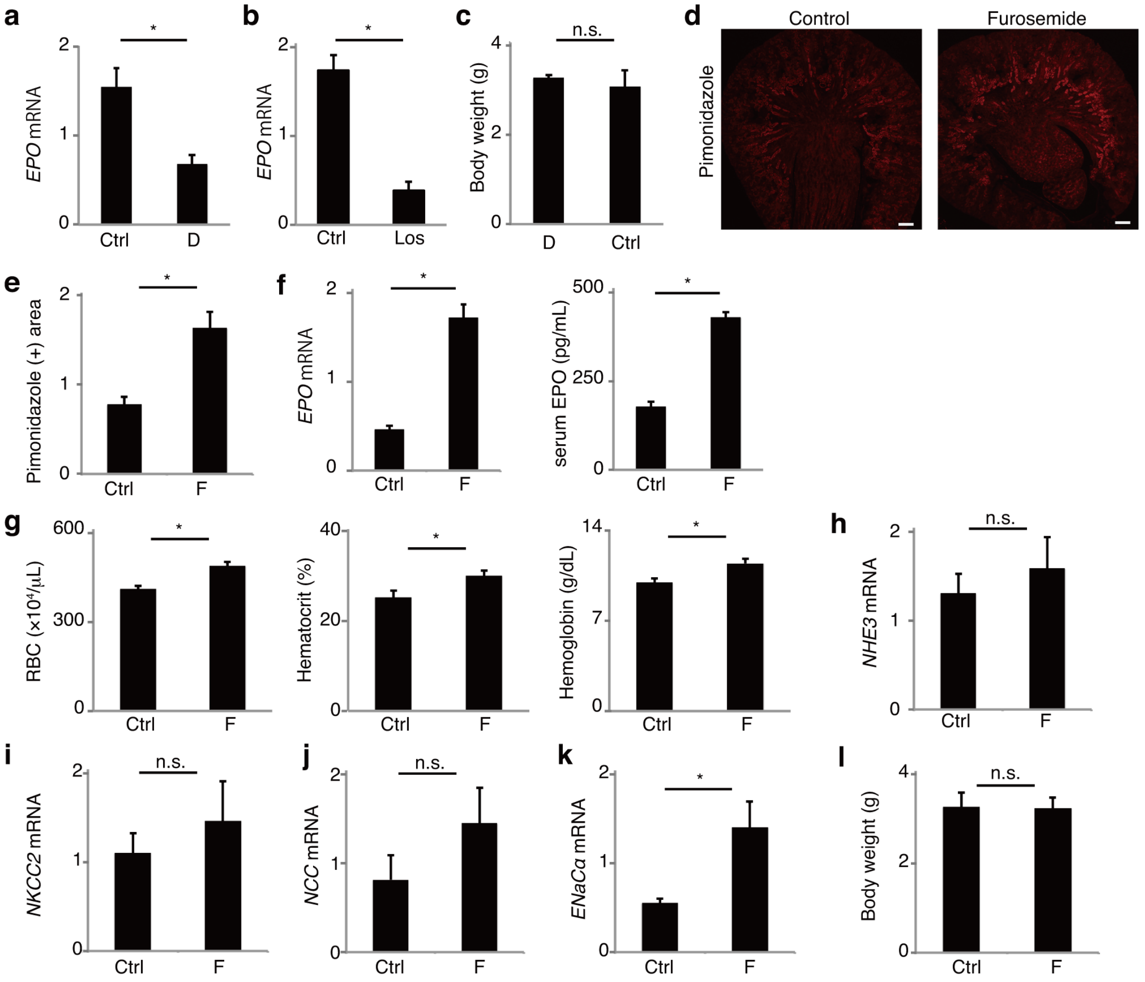

(**a** and **b**) In adult mice, renal *EPO* expression was reduced by short-term use of diuretics (**a**) or losartan (**b**) (n = 4). (**c**) In long-term use of diuretics in neonatal mice from P3 to P7, body weight was comparable between the diuretics group and control (n = 4). (**d-l**) Long-term use of furosemide ameliorated anemia of prematurity. The use of furosemide increased pimonidazole-positive hypoxic area (**d** and **e**), EPO levels (**f**), and parameters of anemia (**g**) (n = 4). Among sodium transporters examined, only *ENaCα* expression was significantly elevated in furosemide group (**h-k**) (n = 4). Body weight was comparable between the two groups (**l**). Error bars indicate SEM. *$P < 0.05$ by t-test. n.s., not significant. Scale bars denote 100 micrometer. Ctrl, control; D, diuretics; Los, losartan; F, furosemide; RBC, red blood cells.

Supplementary figure 4

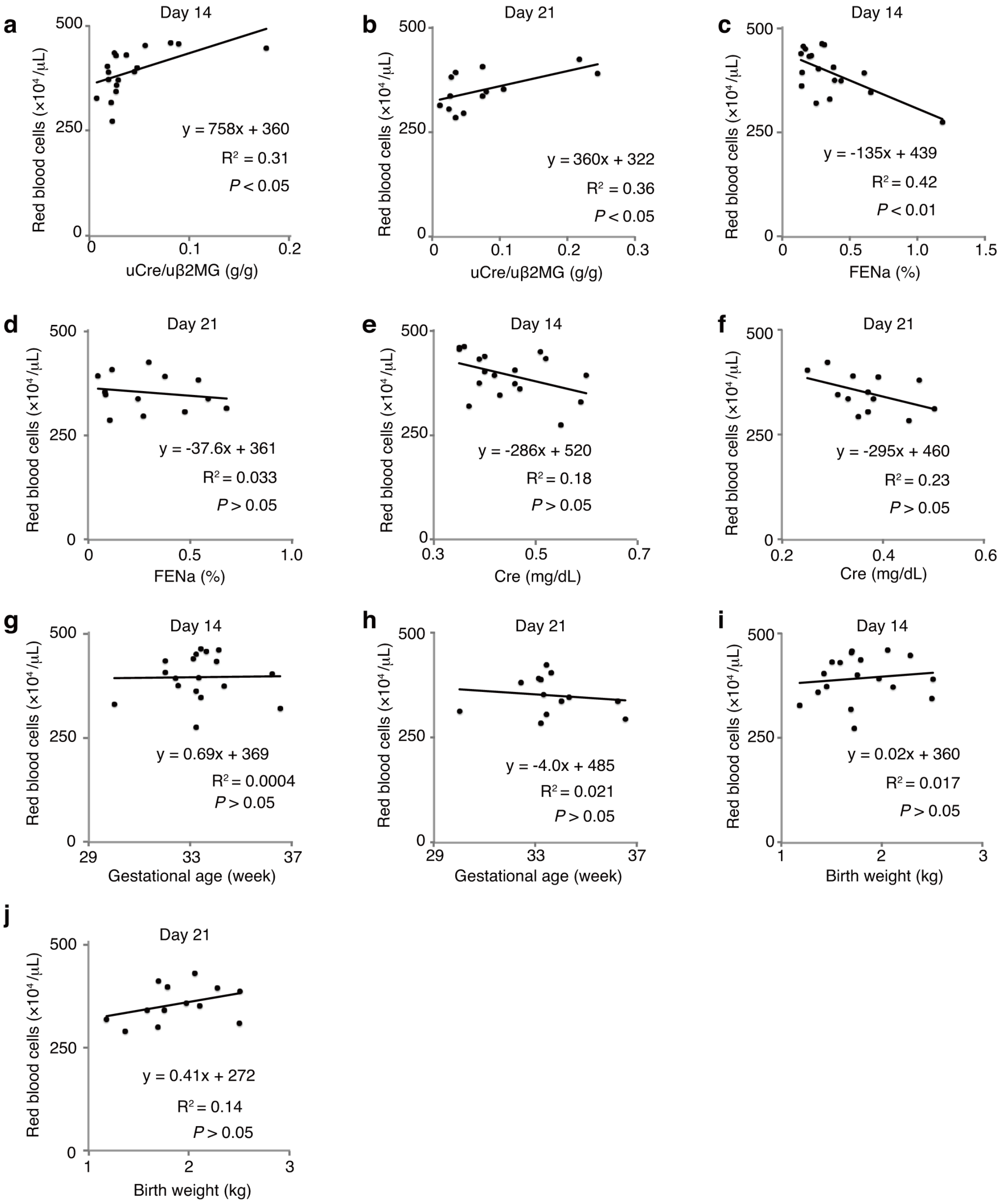

Tubular function correlates with red blood cell counts in preterm neonates. (**a** and **b**) Red blood cell counts were significantly correlated with uCr/uβ2MG at days 14 and 21. (**c** and **d**) Red blood cell counts were correlated with FENa at day 14, but not at day 21. (**e-j**) Red blood cell counts were not correlated with serum creatinine (**e** and **f**), gestational age (**g** and **h**), or birth weight (**i** and **j**). Association between two variables was evaluated by Pearson correlation. *n* = 18 for day 14 and *n* = 13 for day 21. uCr/uβ2MG, urinary creatinine to beta 2-microglobulin ratio; FENa, fractional excretion of sodium; Cre, creatinine.

Supplementary figure 5

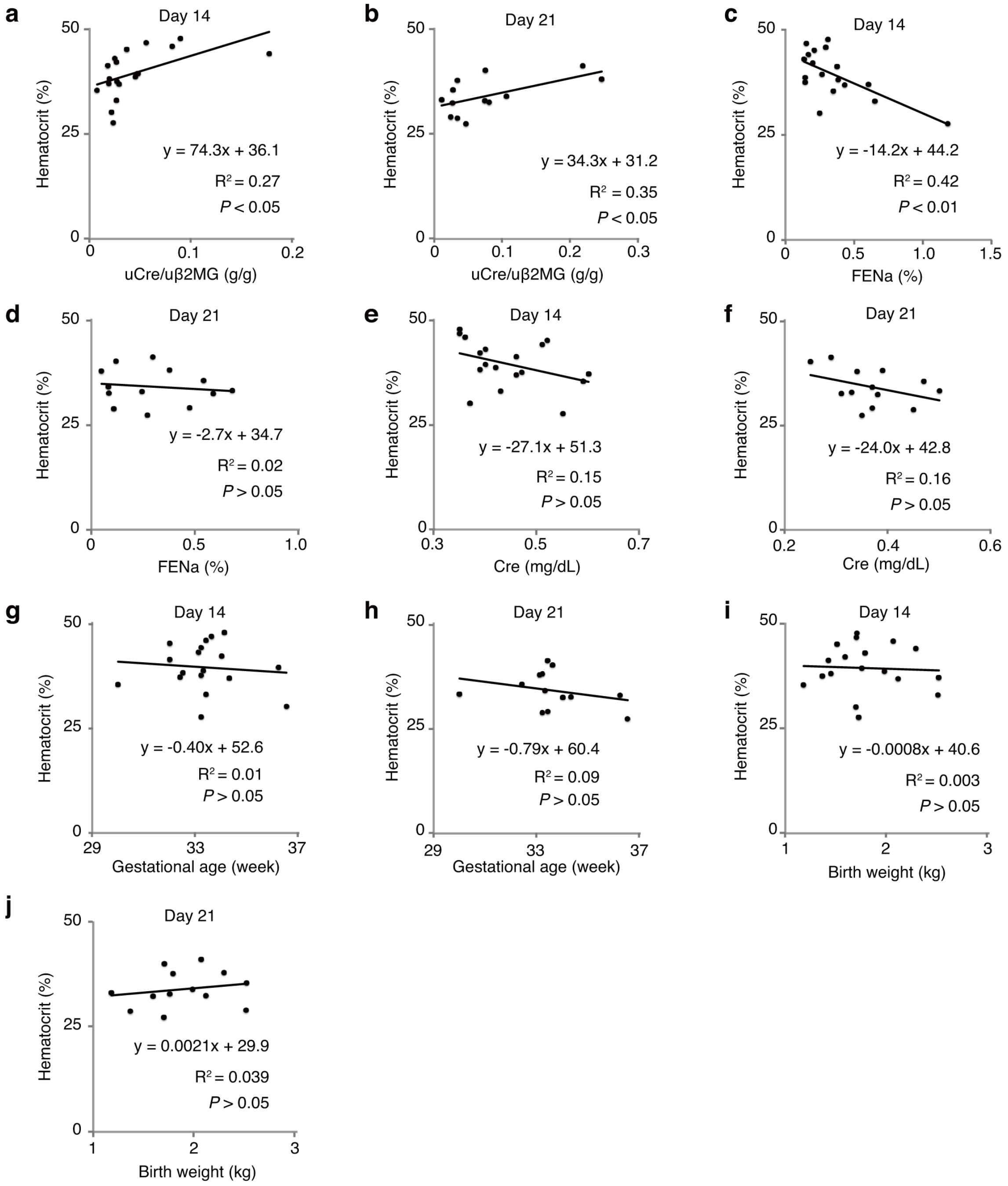

Tubular function correlates with hematocrit levels in preterm neonates. (**a** and **b**) Hematocrit levels were significantly correlated with uCr/uβ2MG at days 14 and 21. (**c** and **d**) Hematocrit levels were correlated with FENa at day 14, but not at day 21. (**e-j**) Hematocrit levels were not correlated with serum creatinine (**e** and **f**), gestational age (**g** and **h**), or birth weight (**i** and **j**). Association between two variables was evaluated by Pearson correlation. n = 18 for day 14 and n = 13 for day 21. uCr/uβ2MG, urinary creatinine to beta 2-microglobulin ratio; FENa, fractional excretion of sodium; Cre, creatinine.
